# Imaging markers of cerebral small vessel disease are associated with Alzheimer’s disease: a systematic review and meta-analysis

**DOI:** 10.3389/fnagi.2025.1498636

**Published:** 2025-01-28

**Authors:** Qi Wu, Jupeng Zhang, Peng Lei, Xiqi Zhu, Changhui Huang

**Affiliations:** ^1^Department of Radiology, Affiliated Hospital of Youjiang Medical University for Nationalities, Baise, China; ^2^School of Testing, Affiliated Hospital of Youjiang Medical University for Nationalities, Baise, China; ^3^Life Science and Clinical Medicine Research Center, Affiliated Hospital of Youjiang Medical University for Nationalities, Baise, China

**Keywords:** cerebral small vessel disease, Alzheimer’s disease, imaging markers, meta-analysis, magnetic resonance imaging

## Abstract

**Objective:**

This study aims to assess the correlation between cerebral small vessel disease (CSVD) imaging markers and the risk of Alzheimer’s disease (AD) through a systematic review and meta-analysis.

**Methods:**

In July 2024, two researchers independently conducted a thorough literature search across databases such as PubMed, Embase, Web of Science, and the Cochrane Library. The selected studies investigated the correlations between white matter hyperintensities (WMHs), cerebral microbleeds (CMBs), lacunar infarction (LI), and enlarged perivascular spaces (EPVS) and the risk of AD. The Newcastle-Ottawa Scale (NOS) was employed to assess the risk of bias in the included cohort and case–control studies.

**Results:**

A total of 6,487 articles were identified, with 15 articles meeting the inclusion criteria. Pooled analyses showed that WMHs (HR: 1.38, 95% CI: 1.10–1.74, *N* = 7,661), CMBs (HR: 1.60, 95% CI: 1.07–2.40, *N* = 6,567), and EPVS (HR: 1.84, 95% CI: 1.24–2.72, *N* = 3,045) were associated with an increased risk of AD, with EPVS showing the strongest correlation. LI did not show a statistically significant association with an increased risk of AD (HR: 1.41, 95% CI: 0.98–2.01, *N* = 4,014).

**Conclusion:**

WMHs, CMBs, and EPVS are associated with an elevated risk of AD, whereas LI is considered a potential risk factor. However, additional studies are required to determine the role of CSVD markers in AD progression.

## 1 Introduction

Alzheimer’s disease (AD) is the most common form of dementia, characterized by progressive cognitive decline and neurodegeneration, accounting for 60 to 80% of all dementia cases (Rostagno, 2022).

Although the pathophysiological processes of Alzheimer’s disease (AD) commence decades before the onset of clinical symptoms, the exact mechanisms driving the disease remain inadequately elucidated (Sperling et al., 2011; Serý et al., 2013; Atri, 2019; Jia et al., 2024).

An increasing body of evidence suggests that vascular factors, particularly those associated with cerebral small vessel disease (CSVD), play a significant role in the development and progression of AD (Hainsworth et al., 2024).

CSVD comprises a variety of disorders affecting small arteries and microvessels of the brain, frequently identified on neuroimaging among elders, manifesting as white matter hyperintensities (WMHs), cerebral microbleeds (CMBs), lacunar infarction (LI) and enlarged perivascular spaces (EPVS) (Liu et al., 2018). These markers have been widely studied and are strongly associated with cognitive impairment and dementia (Shams et al., 2022). WMH refers to specific changes in the white matter that histopathologically reflect myelin pallor, tissue rarefaction associated with loss of myelin and axons, or mild gliosis. WMH were identified as areas of intensive signal on T2-weighted and fluid-attenuated inversion recovery (FLAIR) magnetic resonance imaging (MRI) (Hu et al., 2021). CMBs are small, circular, or elliptical low signals visible on T2-weighted gradient echo sequences and susceptibility-weighted imaging (SWI). CMB was considered a focal deposit of hemosiderin in the brain. LI refers to the pathological changes in the vascular wall of small perforating arteries in the cerebral hemisphere or deep brainstem based on long-term hypertension and arteriosclerosis, causing ischemic softening of brain tissue and forming small infarctions. Finally, EPVS was considered the diameter of perivascular space >2 mm. Compared with other potential markers, such as retinal vascular changes or cerebrospinal fluid biomarkers, these imaging markers offer a comprehensive assessment of different aspects of CSVD pathology, making them particularly suitable for the aims of our study. CSVD has been suggested to be a risk factor for cognitive impairment and dementia. In addition to its role in vascular dementia (VaD), there is a hypothesis suggesting its involvement in the pathogenesis of AD (Inoue et al., 2023).

The relationship between CSVD and AD is complex and bidirectional. While CSVD can contribute to cognitive decline through mechanisms such as chronic hypoperfusion, blood–brain barrier dysfunction, and inflammation, the presence of Alzheimer’s pathology can also exacerbate small vessel disease (Kim et al., 2020; Hainsworth et al., 2024). This interplay suggests that CSVD may not only be a co-occurring pathology but may also serve as a contributing factor in the development and progression of AD. Recent neuroimaging studies have shed light on the potential of CSVD markers as diagnostic tools for AD (Kim et al., 2022).

This study aims to consolidate data from various studies to offer a comprehensive overview of the association between CSVD imaging markers and the risk of AD, thereby contributing to the ongoing endeavors to enhance our understanding and management of AD.

## 2 Methods

This systematic review and meta-analysis was conducted according to the Preferred Reporting Items for a Systematic Review and Meta-analysis (PRISMA) 2020 guidelines (Page et al., 2021). It was registered on the PROSPERO website (CRD42024572219).

### 2.1 Search strategy

In July 2024, two researchers (QW and JZ) independently conducted a systematic search of the electronic databases MEDLINE (PubMed), Cochrane Library, Embase, and Web of Science. In case of disagreement between assessors, consensus was reached through discussion and negotiation. The database coverage was up to July 2024. The language was restricted to English. The full search strategies are available in the Supplementary material.

### 2.2 Inclusion and exclusion criteria

Articles were included based on the satisfaction of all the following criteria: (1) The study subjects were diagnosed with CSVD and confirmed to have no cognitive impairment or only mild cognitive impairment (MCI) through psychological evaluation methods such as the Mini-Mental State Examination (MMSE); simultaneously, they were diagnosed with AD according to the Diagnostic and Statistical Manual of Mental Disorders (DSM) or the Alzheimer’s Disease Diagnostic and Treatment Center criteria. (2) The study design was either cohort or case–control studies; (3) MRI or computed tomography (CT) was required to detect CMB, WMH, LI, or EPVS; (4) all studies must have evaluated the association between any of these four imaging markers and the risk of AD. Exclusion criteria included: (1) subjects with central nervous system disorders other than CSVD; (2) publication types such as review articles, case reports, editorials, conference abstracts, and animal studies; (3) literature that cannot extract risk factors; (4) high dropout rates or follow-up periods not consistent with study design; (5) Newcastle⁃Ottawa Scale (NOS) score < 5 (low-quality literature) (Wells et al., 2013); After the removal of duplicates, titles and abstracts were identified by two independent reviewers using the Covidence systematic review software. Any disagreements were resolved through consensus or arbitration by a third author (XZ).

### 2.3 Literature selection

EndNote 20 software (Clarivate Analytics, Philadelphia, PA, United States) was employed to manage research and remove duplicate projects. Two investigators (QW and JZ) screened the literature based on inclusion and exclusion criteria. Firstly, by reading the titles and abstracts, we eliminated duplicates and those that did not meet the inclusion criteria. Secondly, we read the full text of the literature that may be included and cross-checked the results. Finally, we discussed and negotiated the literature with disputes together. We asked a third reviewer (PL) to further evaluate if consensus could not be reached. For studies with questionable data or missing information, we contacted the authors or corresponding authors to obtain confirmation or supplementation as much as possible. Articles for which missing data could not ultimately be obtained were excluded.

### 2.4 Data extraction

The following data were extracted from each article: study characteristics (name of the first author, publication year, study design, sample size, follow-up periods); population characteristics (country or region, age, sex, baseline cognitive status, and MRI sequence); and result assessments (diagnostic criteria of AD, adjusted factors and risk estimates [RR/HR/OR values and 95% confidence interval (CI)]). If a study only reported OR, particular formulae would transform OR into RR (Xu et al., 2020). If a study fails to report RR/HR, the raw data will be reviewed to see whether RR/HR can be calculated. When the crude and multivariable-adjusted risk estimates were reported simultaneously, the latter would be selected.

### 2.5 Quality assessment

The Newcastle–Ottawa Scale was modified to assess the risk of bias by determining the quality of the selected observational studies (Wells et al., 2013). The scale consists of items divided into three domains: selection, comparison, and exposure (case–control studies) or outcome (cohort studies). The total score is 8 points, with a score of 5 or higher considered high quality. Two independent reviewers (QW and JZ) assessed the study quality, and consensus would be reached through joint reassessment.

### 2.6 Statistical analysis

Review Manager version 5.4.1 (Copenhagen: The Nordic Cochrane Centre, Cochrane Collaboration) and Stata version 15.1 (StataCorp LP, College Station, TX, United States) were used to conduct the meta-analyses. The risk of CSVD imaging markers for AD can be evaluated through the combined HRs and corresponding CIs. The OR value is converted into an RR value according to the formula: RR = OR/[(1−P0) + (P0 × OR)], where P0 is the incidence rate of Alzheimer’s disease in the non-exposed group (i.e., the population without CSVD) (Zhang and Yu, 1998). Higgins inconsistency index (I^2^)-tests were performed to test heterogeneity (Higgins and Thompson, 2002). A fixed-effects model was used when non-significant heterogeneity was observed (*p* > 0.05 and I^2^ < 50%); otherwise (*p* < 0.05 or I^2^ > 50%), a random-effects model was applied, and the source of heterogeneity was analyzed.

In this meta-analysis, we included both studies assessing WMH severity with semiquantitative visual rating scales and studies measuring WMH volume quantitatively. Then subgroup analyses for WMH were conducted according to methods of assessing WMH (volume/the CHS scale/ARWMC scale/Fazekas scale/the Schelten’s scale) and MRI sequences (T2/T2 + FLAIR/T1 + T2 + FLAIR/T2 + FLAIR/T1 + T2). For CMB and LI, we assessed presence versus absence in relation to AD. For EPVS, we evaluated the relationship between their location and AD and performed subgroup analyses. When multiple adjustment models were available for the HR, we included the estimate adjusted for the maximum number of risk factors. Sensitivity analyses were performed after excluding every study to see if the results were stable and to explore the source of heterogeneity. Publication bias was evaluated using Egger’s test, Begg’s test, and funnel plots.

## 3 Results

### 3.1 Literature search

**Figure 1:**
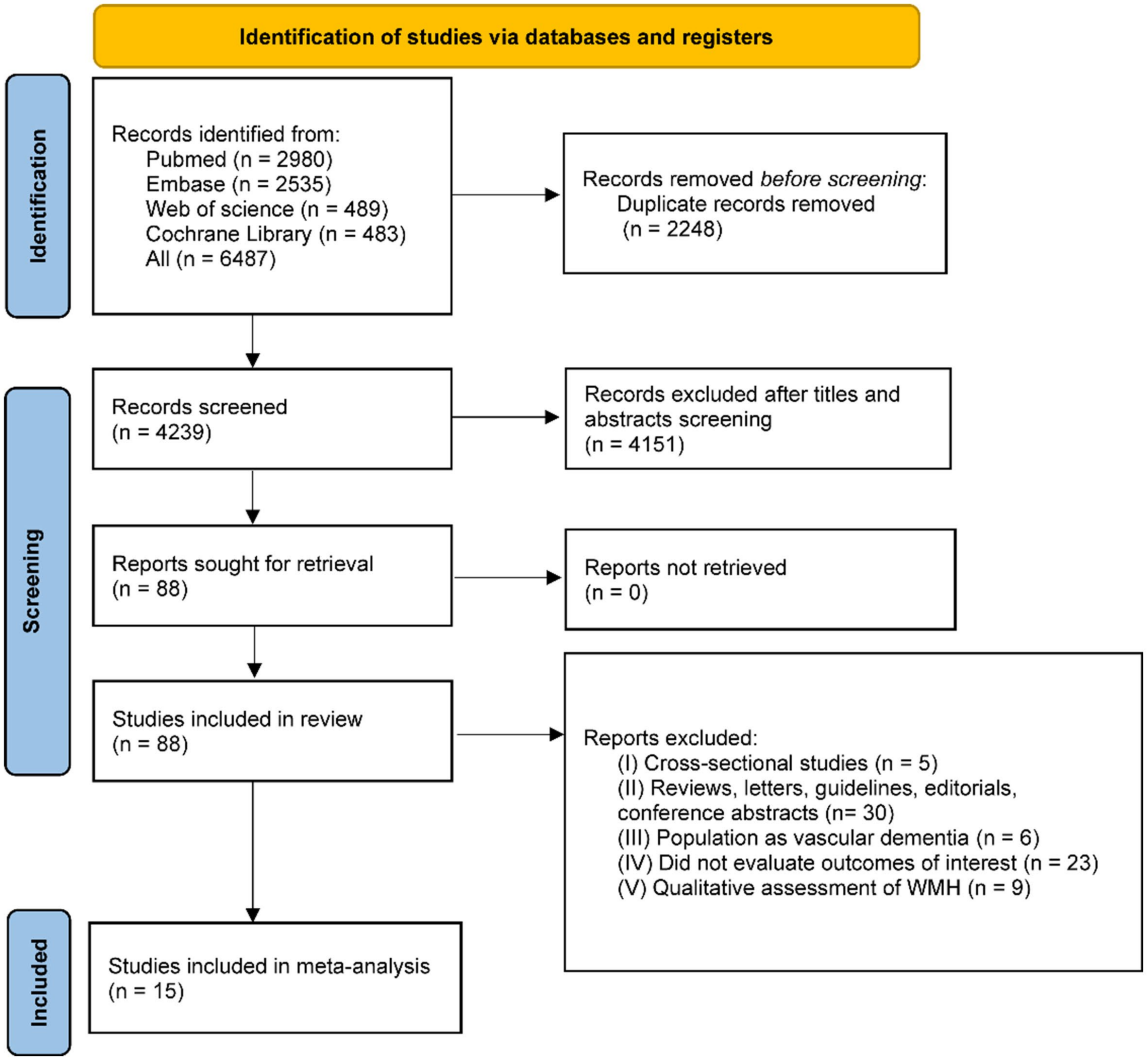
A PRISMA flow chart of the selection procedure. WMH: white matter hyperintensities.

In the initial search, 6,487 records were retrieved. A total of 4,239 articles were obtained after deduplication. Of these, 4,151 articles were excluded as irrelevant after viewing the title and abstract. Then, two researchers (QW and JZ) conducted a full-text review of 88 articles, of which 73 records were excluded, and 15 articles were included in the study. All 15 studies explicitly indicated that individuals with dementia or AD at baseline were excluded from their analyses. The study selection process is shown in the PRISMA flowchart (Figure 1), and the characteristics of the selected studies are shown in Table 1. Based on the Newcastle-Ottawa Quality Assessment Scale, the mean quality score of all included studies was 6.78 (Supplementary Table S1).

**Table 1:** Characteristics of the selected studies.

Author (year)	Study design	Country	Sample size	Age, year	Sex (female %)	MRI sequence	Follow-up Periods	HR/RR/OR	Definition of AD	Adjusted factors
Romero et al. (2017)	Cohort	America	1,296	72.0 ± 8.0	700(54.0)	T1; T2-GRE	6.7 ± 2.7	HR: CMBs: 2.30(1.16–4.55)	DSM-IV, NINCDS	age, sex, educational level, APOEε4, hypertension, diabetes CVD
Chen et al. (2022)	Cohort	China	137	81.0 ± 4.5	88(64.2)	SWI; T1; T2; FLAIR; TOF MRA	NA	OR: WMH: 2.62(1.23–5.57); CSO PVSE: 1.45 (0.84–2.50); BG PVSE:1.03 (0.57–1.87)	NIA-AA, CDR	age, sex, and vascular risk factors, CDR
Ding et al. (2018)	Cohort	China	278	68.6 ± 6.0	148(53.2)	T1; T2 FLAIR; GRE	6	RR: WMH: 3.16(1.50–6.64); LI: 2.73(1.21–6.15); CMBs:1.16(0.34–4.02)	DSM-IV	age, sex, education, baseline MMSE, history of hypertension, DM, hyperlipidemia, AF, CAD, APOEε4
Miwa et al. (2016)	Cohort	Japan	803	67.0 ± 8.5	330(41.1)	NA	7.5 ± 3.2	RR: WMH: 0.99(0.91–1.06); LI: 0.85(0.32–2.18)	NINCDS⁃ADRDA	age, sex, education level, APOEε4
Ye et al. (2019)	Cohort	China	2,626	68.6 ± 8.0	1,161(51.2),194 (54.3)	T1; T2 FLAIR	5	HR: WMH: 1.40(1.25–1.59); LI:1.23 (1.14–1.39)	NINDS-AIREN	age, sex, education level, hypertension, hyperlipidemia, diabetes, smoking status, alcohol consumption status, ApoE level
Romero et al. (2022)	Cohort	America	1,449	67.5 ± 9.8	785(54.2)	T1; T2	8.3	HR: CSO EPVS: 5.34 (2.72, 10.45); BG EPVS:2.20 (0.66, 7.34)	DSM-IV, NINCDS-ADRDA	age, sex, time between MRI and examination cycle, education, vascular factors, CVD
Paradise et al. (2021)	Cohort	Australia	414	79.8 ± 4.6	218(52.7)	SWI; T1; T2; FLAIR	8	OR: CSO EPVS: 1.42(0.84–2.38); BG EPVS: 1.46(0.86–2.46)	NA	Age; Sex; Education; APOEε4; carrier status; BMI; Smoking status; hypertension; diabetes neuroimaging measures
Shi et al. (2023)	Cohort	China	1,045	43.1 ± 9.9	496(47.4)	T1; T2; SWI; FLAIR; DWI	14	OR: CSO EPVS: 3.57 (0.31–41.59); BG EEPVS: 2.56(0.97–6.77)	MoCA	sex, baseline age, drinking, smoking, physical exercise, medication history, average fasting blood glucose, BMI, use of hypoglycemic and hypolipidemic medications period, education level
Eckerström et al. (2015)	Cohort	Sweden	101	66.2 ± 7.0	64(63.4)	T2	3.0 ± 1.8	HR: WMH: 0.5(0.06–3.8)	NINCDS-ADRDA	NA
Akoudad et al. (2016)	Cohort	Netherland	4,841	63.8 ± 10.9	2,663(55.0)	T1; T2; FLAIR; PD	4.8 ± 1.4	HR: CMBs:1.67(0.83–3.36)	DSM⁃III⁃R, NINCDS⁃ADRDA	Age, sex, education level, ApoEε4, hypertension, high-density lipoprotein cholesterol, smoking, diabetes, lipid drug taking, anticoagulant therapy
Miwa et al. (2014)	Cohort	Japan	524	67.70 ± 8.30	222(42.4)	T1; T2; FLAIR; T2-GRE	7.5	HR CMBs: 1.22(0.32–3.72)	DSM⁃III⁃R, MMSE, CDR;	Age, education degree, APOEε4
Staekenborg et al. (2009)	Cohort	Nederland	152	70.0 ± 6.0	71(46.7)	T1; T2 FLAIR	2.0 ± 1.0	HR: WMH:1.20(0.70–2.20); LI:1.10(0.50–2.20); CMBs:0.80(0.20–2.20)	NINCDS⁃ADRDA	Age, sex
Rosano et al. (2007)	Case–control	America	155	77.4 ± 3.4	93(60.0)	T1; T2; SPGR	NA	OR: WMH:0.8(0.30–2.80); LI:2.7(1.00–7.10)	NINCDS⁃ADRDA, AADDTC	Age, sex, race, education degree, MMSE score, ApoEε4, CVD
Keller et al. (2023)	Cohort	Iceland	3,077	75.6 ± 5.2	1900(61.7)	T1; FLAIR	9.9 ± 2.6	HR: WMH:1.68 (1.54–1.87)	NA	Age, sex, cognitive status at baseline, intracranial volume, BMI, hypertension, diabetes mellitus, cholesterol level, smoking status
Tosto et al. (2014)	Cohort	America	332	74.6 ± 7.4	113 (34.04)	T1; T2; PD	4	HR: WMH:1.23(1.05–1.43)	NA	Age, sex, APOEε4, education degree

NA, not accessible; WMH, white matter hyperintensities; AD, Alzheimer’s disease; CVD, cardiovascular disease; ApoEε4, apolipoprotein E ε4; MRI, Magnetic Resonance Imaging; BMI, body mass index; T1, T1 weight; T2, T2 weighted image; FLAIR, fluid attenuated inversion recovery; PD: proton density–weighted; GRE: gradient recall echo sequences; SPGR: spoiled gradient recalled acquisition; AD, Alzheimer’s disease; CMBs, cerebral microbleeds; LI, lacunar infarct; WML, white matter lesion; CSO EPVS: enlarged perivascular spaces in the centrum semiovale; BG EPVS: enlarged perivascular spaces in the basal ganglia; HR, hazard ratio; RR, relative risk; OR, odds ratio; DSM-IV, Diagnostic and Statistical Manual of Mental Disorders Fourth Edition; NINCDS-ADRDA, National Institute of Neurological and Communicative Disorders and Stroke and Alzheimer’s Disease and Related Disorders Association Alzheimer’s Criteria; NIA-AA, criteria of the National Institute on Aging /Alzheimer’s Association workgroup; NIHR, National Institute for 97 Health Research; NINDS-AIREN, the National Institute of Neurological Disorders and Stroke-the Association Internationale pour la Recherche et 1′Enseignement en Neurosciences diagnostic criteria; ADDTC, Alzheimer’s Disease Diagnostic and Treatment Centers Criteria; DSM-III-R, Diagnostic and Statistical Manual of Mental Disorders, 3rd edition, revise; MMSE, Mini-mental State Examination; CDR, The Clinical Dementia Rating.

### 3.2 WMHs and risk of AD

**Figure 2:**
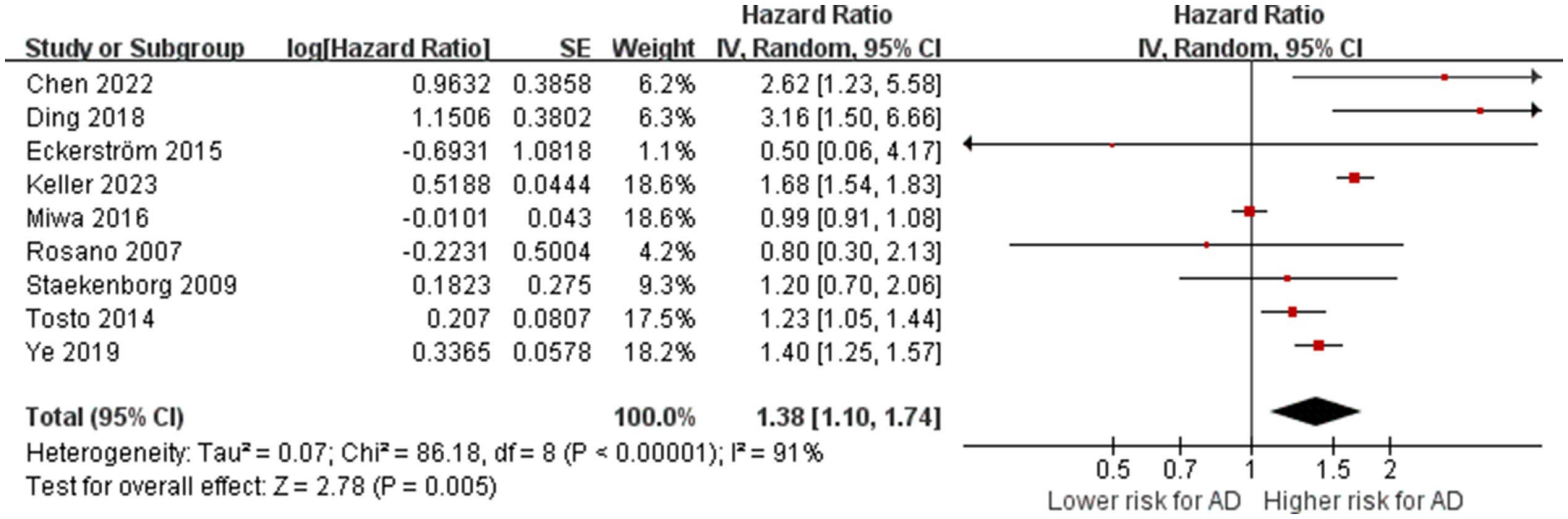
Association between WMHs with AD. IV = inverse variance. AD = Alzheimer’s disease; CI = confidence interval.

A total of nine studies with 7,661 participants investigated the relationship between WMH volume and AD (Rosano et al., 2007; Staekenborg et al., 2009; Tosto et al., 2014; Eckerström et al., 2015; Miwa et al., 2016; Ding et al., 2018; Ye et al., 2019; Chen et al., 2022; Keller et al., 2023). There was substantial heterogeneity between studies (*p* < 0.01, I^2^ = 91%), so a random-effects model was used for the combined-effects analysis. The meta-analysis indicated that cerebral WMH is associated with an increased risk of AD (HR = 1.38, 95% CI: 1.10–1.74, *p* = 0.005; Figure 2), suggesting that cerebral WMH is a risk factor for AD.

Table 2 summarizes the results of the subgroup analysis for AD. 8 studies were used for subgroup analysis, as the remaining one did not provide a specific evaluation method for WMH (Miwa et al., 2016). The subgroup analysis based on MRI sequences demonstrated the association between WMH with AD remained significant on T2-weighted images combined with FLAIR sequence (HR = 2.62, 95% CI = 1.23–5.58; one study), T1-weighted images, T2-weighted images combined with FLAIR sequence (HR = 1.57, 95% CI = 1.05–2.33; 3 studies), T1-weighted images combined with FLAIR sequence (HR = 1.68, 95% CI = 1.54–1.83; one study), and T1-weighted images combined with T2-weighted images (HR = 1.22, 95% CI = 1.04–1.42; two studies). However, the study rating WMH by simply T2-weighted images showed a neutral result (HR = 1.22, 95% CI = 1.04–1.42; one study). The difference between subgroups is statistically significant (*p* = 0.07). Studies showed a positive association of WMH volume with AD (HR =1.45, 95% CI = 1.07–1.96; 2 studies). Meta-analyses of WMH severity assessed by two semiquantitative visual scales showed significant results (ARWMC scale, HR = 2.88, 95% CI = 1.69–4.90, 2 studies; Fazekas scale, HR = 1.40, 95% CI = 1.25–1.56, 2 studies), while the other two scales showed negative results (the CHS scale, HR = 0.80, 95% CI = 0.30, 2.13, one study; the Schelten’s scale, HR = 1.20, 95% CI = 0.70–2.06, one study). The difference between subgroups is significant (*p* = 0.004). Subgroup analyses suggested that substantial heterogeneity among studies on WMH may be due to the inconsistency of assessment methods and MRI sequences. Forest plots of the above results are presented in the Supplementary Figures S5, S6.

**Table 2:** Association between WMH and AD according to subgroups.

Subgroups	No. of studies	Pooled HR (95%CI)	*P* value	I^2^ (%)
Overall	8	1.47 (1.24, 1.75)	*p* = 0.002	69.7
Assessment methods				
Volume	2	1.45 (1.07, 1.96)	*p* = 0.017	91.3
CHS scale	1	0.80 (0.30, 2.13)	*p* = 0.656	0
ARWMC scale	2	2.88 (1.69, 4.90)	*P* < 0.01	0
Fazekas scale	2	1.40 (1.25, 1.56)	*P* < 0.01	0
Schelten’s scale	1	1.20 (0.70, 2.06)	*p* = 0.507	0
MRI sequences				
T2	1	0.50 (0.06, 4.17)	*p* = 0.522	0
T2 + FLAIR	1	2.62 (1.23, 5.58)	*p* = 0.013	0
T1+ T2 + FLAIR	3	1.57 (1.05, 2.33)	*p* = 0.028	58.8
T1 + FLAIR	1	1.68 (1.54,1.83)	*P* < 0.01	0
T1 + T2	2	1.22 (1.04, 1.42)	*p* = 0.014	0

AD = Alzheimer disease, HR = hazard ratio, MRI, Magnetic Resonance Imaging; T1, T1 weighted image; T2, T2 weighted image; FLAIR, fluid-attenuated inversion recovery; Weighted; WMH, white matter hyperintensities; CHS, the Cardiovascular Health Study; ARWMC, the age-related white matter changes.

Among these nine studies, significant associations of periventricular WMH (HR = 2.62, 95% CI: 1.23–5.57) and deep WMH (HR = 1.65, 95% CI: 0.93–2.96) with AD subtypes were provided in only one study (Chen et al., 2022).

### 3.3 CMBs and risk of AD

**Figure 3:**
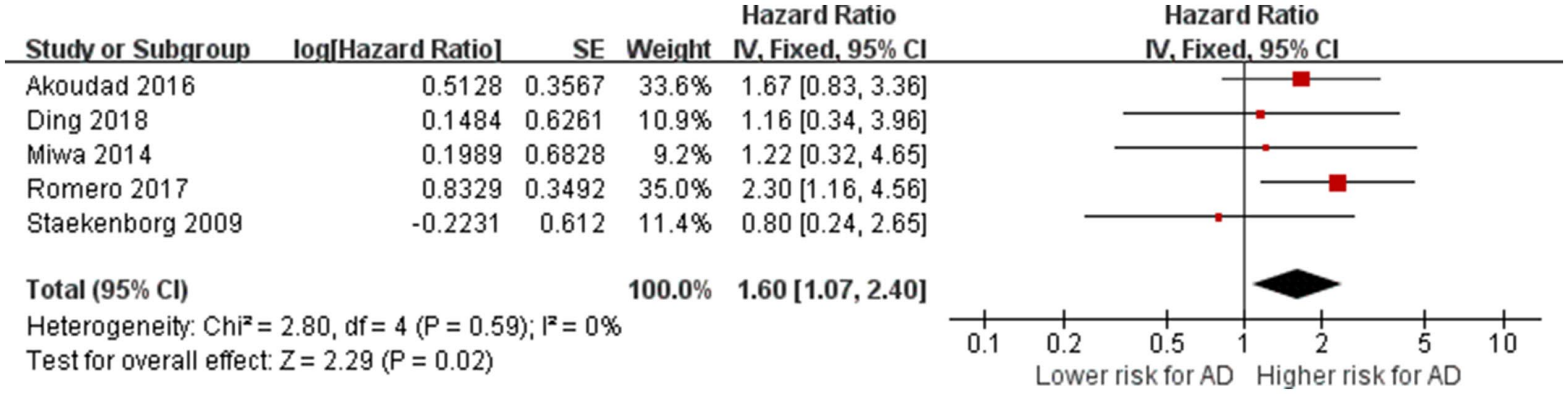
Association between CMBs and AD. IV = inverse variance. AD = Alzheimer’s disease; CI = confidence interval.

A total of five studies with 6,567 participants explored the relationship between the presence of CMBs and the risk of AD (Staekenborg et al., 2009; Miwa et al., 2014; Akoudad et al., 2016; Romero et al., 2017; Ding et al., 2018). After meta-analyzing the effect estimates of these studies, we found no heterogeneity between studies (*p* = 0.59, I^2^ = 0%). The meta-analysis showed that the presence of CMBs is associated with an increased risk of AD (HR = 1.60, 95% CI: 1.70–2.40, *p* = 0.02; Figure 3), suggesting that the presence of CMBs is a risk factor for AD.

### 3.4 Lacunar infarction and risk of AD

**Figure 4:**
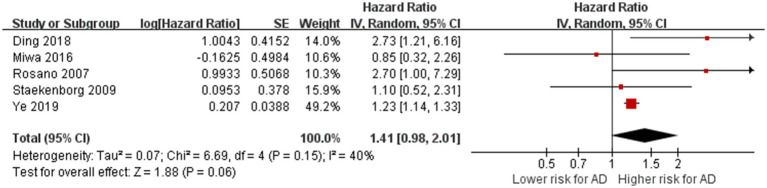
Association between lacunar infarction and AD. IV = inverse variance. AD = Alzheimer’s disease; CI = confidence interval.

Five studies reported on the relationship between LI and the risk of AD (Rosano et al., 2007; Staekenborg et al., 2009; Miwa et al., 2016; Ding et al., 2018; Ye et al., 2019). The total number of participants was 4,014. After pooling the reported effect estimates of these studies, we found no heterogeneity between studies (*p* = 0.15, I^2^ = 40%). The meta-analysis indicated that LI did not increase the risk of AD (HR = 1.41, 95% CI: 0.98–2.01, *p* = 0.06; Figure 4), indicating that lacunar infarcts are not statistically a risk factor for AD.

### 3.5 EPVS and risk of AD

**Figure 5:**
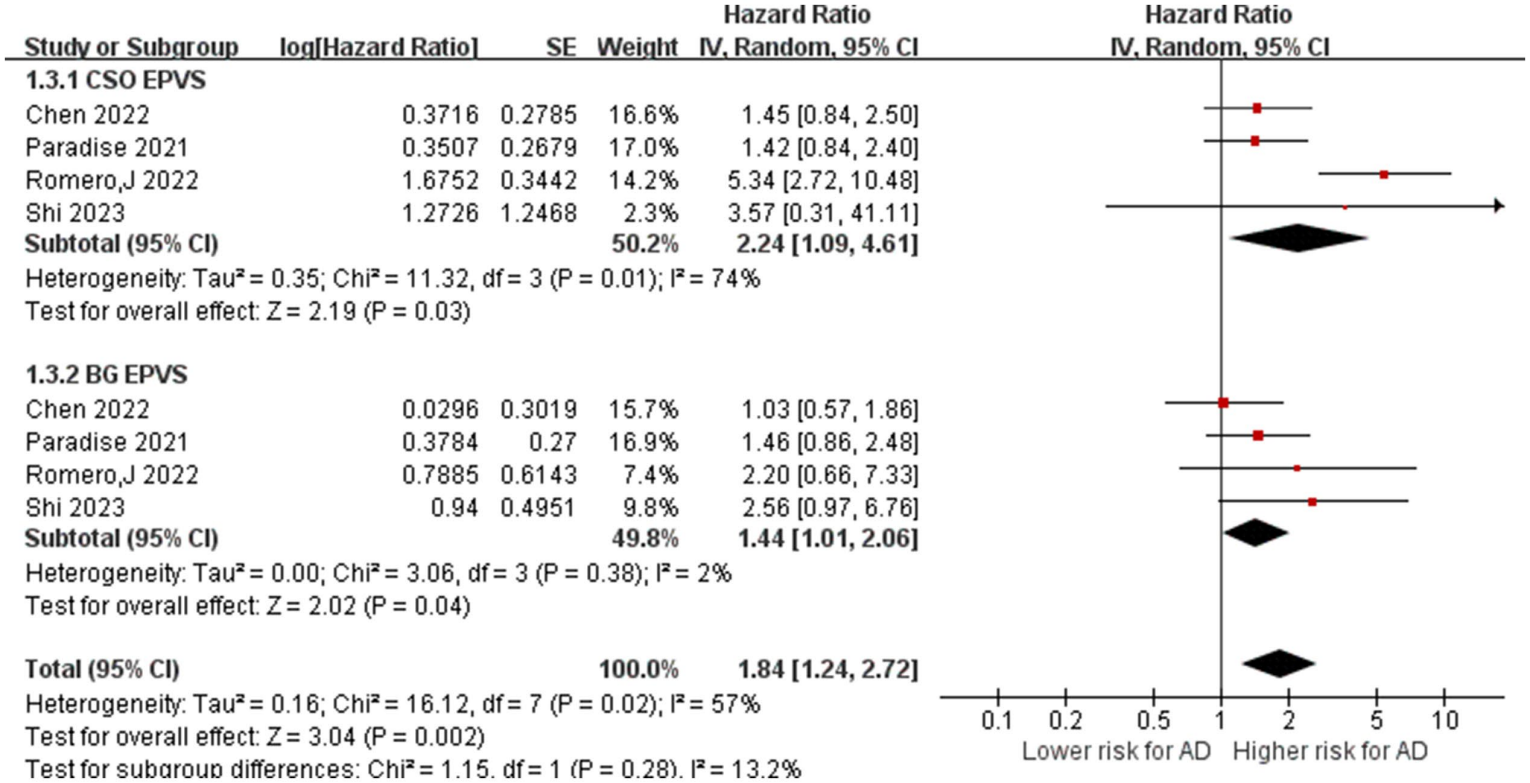
Association between EPVS and AD. IV = inverse variance. AD = Alzheimer’s disease; CI = confidence interval.

A total of four studies with 3,045 participants investigated the relationship between EPVS and AD (Paradise et al., 2021; Chen et al., 2022; Romero et al., 2022; Shi et al., 2023). These four studies evaluated EPVS in the central semiovale (CSO-EPVS) and EPVS in the basal ganglia (BG-EPVS) concerning the risk of AD. For CSO-EPVS, the pooled HR was 2.24 (95% CI: 1.09–4.60, *p* = 0.03), respectively, with high heterogeneity (I^2^ = 74%, *p* = 0.01). For BG-EPVS, the pooled accuracy was 1.44 (95% CI: 1.01–2.06), respectively, with no heterogeneity (I^2^ = 2%, *p* = 0.38). Finally, the inter-group heterogeneity was high (I^2^ = 57%, *p* = 0.02), indicating that the risk of AD was different with different locations, with CSO-EPVS having a higher risk of AD than BG-EPVS. The subgroup analyses are presented in Figure 5.

### 3.6 Sensitivity analyses

The sensitivity analysis, excluding studies one by one, showed that, except for LI, the meta-analysis results of other imaging markers were stable and reliable (Supplementary Figures S1–S4). The heterogeneity of AD risk in patients with WMH may stem from the different baseline cognitive status of the included patients, not adjusted for measures of focal or total gray matter volume or head size, inconsistent imaging diagnostic criteria, and varying follow-up times. Notably, different studies exhibit considerable variation in their assessment of WMHs, encompassing both qualitative and quantitative evaluations and employing diverse MRI sequences to quantify these hyperintensities.

### 3.7 Publication bias

**Figure 6:**
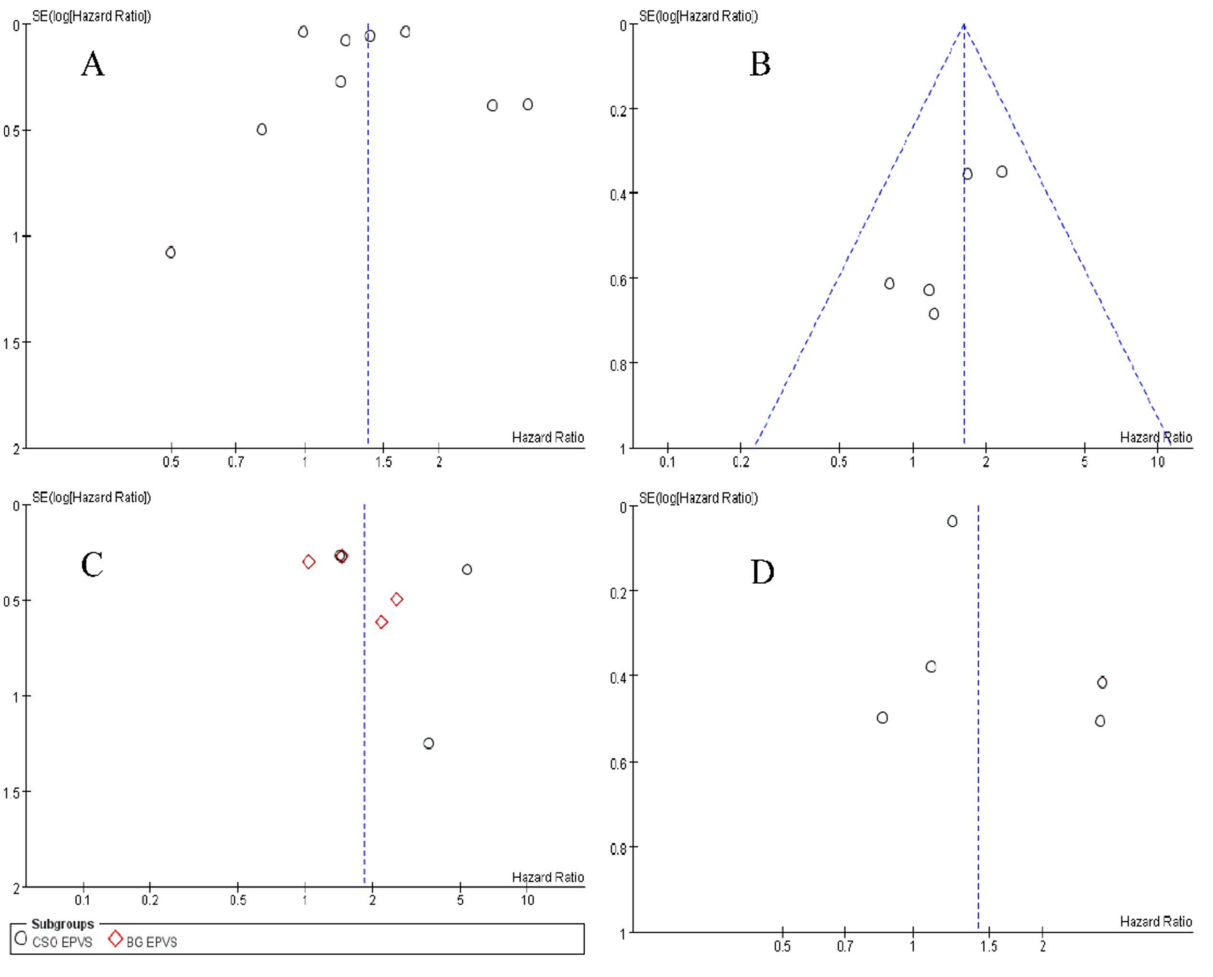
Funnel plot of WMHs **(A)**; CMBs **(B)**; lacunar infarction **(C)**; EPVS **(D)**.

Begg’s and Egger’s tests indicate that the literature on the risk of AD among patients with WMHs, CMBs, EPVS, and LI exhibit no publication bias, as shown in Table 3. The funnel plots are symmetrically distributed (Figures 6A–D), implying no publication bias in the included studies.

**Table 3:** Publication bias of different kinds of literature reporting imaging markers of CSVD associated with AD.

Imaging markers	Begg’s test		Egger’s test	
	*Z*	*P*	*t*	*P*
WMH	0.30	0.754	0.26	0.800
CMB	0.73	0.462	−2.73	0.072
LI	0.73	0.462	0.91	0.431
EPVS	1.11	0.266	1.05	0.334

WMH: white matter hyperintensities; CMB: cerebral microbleeds; LI: lacunar infarction; EPVS: enlarged perivascular spaces.

## 4 Discussion

This study systematically collected clinical research on the correlation between CSVD and AD and conducted a meta-analysis. We found that WMHs, CMBs, and EPVS are important indicators of higher risk for AD, while LI is a potential risk indicator for AD.

### 4.1 White matter hyperintensities

Our research was in line with the recent meta-analyses, which suggested WMH increases the risks of cognitive impairment and AD (Hu et al., 2021). Various MRI sequences have been employed to explore the association between WMH and dementia (Mortamais et al., 2014; Mustapha et al., 2019), and our results of sub-analyses conducted through MRI sequences suggested the most significant association of WMH with AD on T2-weighted images combined with FLAIR sequence (HR = 2.62, 95% CI = 1.23–5.58). This may be attributed to the fact that T2-weighted images and FLAIR sequences are generally more sensitive in detecting WMH, particularly those caused by chronic ischemic changes, while T1-weighted images can provide complementary information about atrophy and the integrity of brain structures (Wardlaw et al., 2013). Meanwhile, a non-significant result was shown when WMH was assessed using CHS and Schelten’s scale. The possible reason was that the low sensitivity and limited maximum boundary of the CHS scale and Schelten’s scale might limit the detectability of WMH and lead to underestimation of the relative risk for AD (Hu et al., 2021). It should be emphasized that a particular difference is the relatively small number of Asians and Africans (40%) included in this study. In addition to the well-known influences of age and gender, preliminary evidence suggests that there may be significant differences in the burden of WMH across different races, which could also partly explain the high heterogeneity between studies (Nyquist et al., 2014). A more widely accepted method of assessing WMH should be proposed in future studies. In this analysis, we incorporated a study by Rosano et al. (2007), which indicated that the presence of WMH is not associated with the onset of AD, while the location of the WMH may lead to the development of AD. WMH is most commonly found in the periventricular and subcortical regions, which are associated with cognitive decline, especially in executive functions. In contrast, deep white matter lesions have a more direct effect on processing speed and attention. Kim et al. also believe that the risk of AD associated with periventricular white matter lesions is significantly higher than in other areas (Kim et al., 2015). It has been demonstrated that the distribution of WMH determines the characteristics of cognitive impairments (Biesbroek et al., 2016).

Pathological changes of WMH included gliosis, axonal loss, and ischemic demyelination. The association between WMH and cortical vascular amyloid beta (Aβ) is still debated, with conflicting findings (Graff-Radford et al., 2019; Sweeney et al., 2019). Some studies suggest that WMH and cortical Aβ are independent and additive pathological processes leading to cognitive deficits, while others propose that the lesions interact and synergistically affect cognition (Dupont et al., 2020; Garnier-Crussard et al., 2020). Longitudinal studies have also suggested that WMHs could not cause Aβ deposition but could alter Aβ pathology (Ortega et al., 2021). Some studies have found that WMH can predict tau pathology, indicating that regional distribution of WMH affects AD by enhancing the impact of tau on clinical outcomes (Brickman et al., 2015; Alban et al., 2023). However, in a study focusing on whether WMH volume moderates the independent association between AD biomarkers and cognition, linear regression was used to test that there was no interaction between WMH volume and tau PET in cognitive measurement (Edwards et al., 2023).

### 4.2 Cerebral microbleeds

Microbleeds in patients with AD are associated with cerebral amyloid angiopathy (CAA) due to Aβ deposits (Ikeda et al., 2021). It is related to the decrease in Aβ_42_ content in cerebrospinal fluid of patients with CMB and AD, indicating that the efflux of Aβ_42_ is affected in patients with CMBs, and more Aβ_42_ is deposited in cells. Moreover, ApoE ε4 may also be one of its influencing factors (Kalaria and Sepulveda-Falla, 2021). Vázquez-Justes et al. enrolled 98 patients diagnosed with mild to moderate AD and examined AD cerebrospinal fluid (CSF) biomarkers and APOE genotypes at the initial assessment. Their investigation revealed that the presence of cerebral amyloidosis and the APOE ε4 allele correlated with CMBs (Vázquez-Justes et al., 2022). Our analysis corroborates the preceding research outcomes, indicating that CMB contributes to the onset and progression of AD.

In the context of the relationship between microbleeds and AD, it is worth noting that the number and location of microbleeds were not considered, which could be more important than merely assessing their presence or absence. Akoudad and colleagues discovered that three or more microbleeds were significantly correlated with cognitive impairment (Akoudad et al., 2016). Studies have shown that periventricular microbleeds have a more significant association with Aβ deposition than other regions (Goos et al., 2009). However, it is intriguing that the location of microbleeds (i.e., whether they are in the lobes, deep, or infratentorial regions) may not impact their association with dementia (Romero et al., 2017). This partially contrasts the prevailing viewpoint that lobar-specific CMBs are primarily linked to AD (Sheikh-Bahaei et al., 2019; Yatawara et al., 2020).

### 4.3 Lacunar infarction

The pathological relationship between LI and AD remains unclear. While the impacts of WHM and CMB on broader AD-related neurodegeneration and amyloid or tau pathology may be less pronounced, LI appears to exert a more localized effect on cognitive function without significantly affecting global brain atrophy or AD-specific biomarkers. Studies have shown that LI is not associated with changes in CSF Aβ levels but does show a positive correlation with plasma Aβ levels (Liu et al., 2018; Jiang et al., 2021). LI frequently occurs in the basal ganglia and thalamus, impairing motor control and contributing to executive dysfunction and memory issues. The risk of developing AD is greater when LIs are located in the basal ganglia compared to other areas. Additionally, the number of small infarcts is directly related to the severity of cognitive decline (Bos et al., 2018). Infarcts in white matter-rich areas or near regions affected by amyloid and tau deposits can accelerate AD progression (Wanggong et al., 2021).

However, the overall effect observed in our meta-analysis was not statistically significant. The lack of association between LI and AD may be explained by the small number of clinical trials and variations in lesion location, highlighting the need for further validation through high-quality, large-sample cohort studies. Additionally, the inconsistency in terms used to describe similar conditions—such as lacunes, subcortical infarcts, lacunar strokes, and microinfarcts—complicates comparisons across studies, leading to varying conclusions (Jiang et al., 2021). Notably, most studies included in our meta-analysis did not use FLAIR sequences to identify silent cerebral infarctions, relying instead on conventional T1 or T2-weighted sequences. This approach increases the likelihood of misclassifying EPVS as LIs, which may contribute to the observed lack of a significant correlation between LI and AD.

### 4.4 EPVS

In the context of CSVD, potentially important novel imaging markers have been identified, namely cortical microinfarcts and EPVS (Lynch et al., 2022). Increasing evidence suggests that EPVS is linked with Aβ deposition, particularly in cortical and hippocampal regions. EPVS may contribute to Aβ accumulation by impairing the brain’s waste clearance processes (Gertje et al., 2021). Although less research has focused on the interaction between EPVS and tau pathology, some evidence suggests that EPVS creates an environment that facilitates tau accumulation by compromising the blood–brain barrier and inducing neuroinflammation (Sibilia et al., 2023).

Notably, we found that CSO-EPVS (HR: 2.24, 95% CI: 1.09–4.60) showed a stronger correlation with AD than BG-EPVS (HR: 1.44, 95% CI: 1.01–2.06), indicating that the risk of AD may vary depending on the location of EPVS. The findings of Jung et al. support the hypothesis that BG-EPVS is associated with CSVD and vascular risk factors, whereas CSO-EPVS is associated with cerebral amyloid angiopathy (Jung et al., 2024). Aside from the location of perivascular space, recent research has explored whether perivascular space volume is related to dementia. Interestingly, in the research of Hong et al., it was found that changes in BG-PVS (HR: 0.75, 95% CI: 0.38–1.44) and white matter perivascular space (WM-PVS) (HR: 1.13, 95% CI: 0.70–1.83) volumes did not predict dementia conversion during a 5-year follow-up (Hong et al., 2024).

### 4.5 Strengths and limitations

This study has several strengths. Firstly, to avoid differences in meta-analysis results caused by inconsistencies between studies, subgroup analyses were conducted for different rating scales and MRI sequences of WMH. Secondly, compared to previous meta-analyses, our review included additional studies on the relation between EPVS and the risk of AD and performed subgroup analysis based on its occurrence location. We also used funnel plot symmetry to evaluate publication bias. Finally, we focused on appraising the available research evidence on the association between imaging markers of CSVD and AD rather than dementia and explored potential sources of heterogeneity in more detail.

This study also has certain limitations. First, despite conducting subgroup analyses to explore the association between WMH and the risk of AD, some sub-analyses still exhibited heterogeneity. This issue may be attributed to the relatively small number of studies included, highlighting the need for further validation through high-quality, large-sample cohort studies. Second, the potential impact of covariates such as age, sex, APOE haplotype, and CDR score on the CSVD-AD relationship was not fully explored in this meta-analysis. Future studies should consider these covariates in their analyses.

Furthermore, the absence of a detailed analysis regarding the influence of various imaging diagnostic criteria, lesion locations, and severity levels may introduce bias into the results. Thus, the integration of resources from multicenter collaborations using standardized MRI protocols and harmonized methodologies can provide further insights into the role of CSVD in the etiology of AD. While this study focused on the association between a single neuroimaging biomarker of CSVD and AD, previous studies have shown a dynamic interplay among microbleeds, lacunar infarction, white matter hyperintensities, and perivascular spatial expansion (Wardlaw et al., 2017; Ohlmeier et al., 2023).

Finally, in addition to the biomarkers discussed in our article, other indicators of CSVD (such as subcortical microinfarction, the total load of CSVD, and brain atrophy) have varying degrees of association with cognitive decline and AD. However, due to the lack of sufficient evidence, these associations were not confirmed in this meta-analysis.

## 5 Conclusion

In summary, we found that WMHs, CMBs, and EPVS are associated with an increased risk of AD. In addition, LI emerged as a potential risk factor for AD. These findings suggest that CSVD plays a role, to some extent, in the onset and progression of AD. Therefore, primary prevention of AD should be considered for individuals exhibiting these CSVD symptoms on imaging scans. However, our findings highlight that the data linking imaging markers of CSVD to AD are still limited. Further research is required to clarify their exact role in the etiology of dementia.
