# Supplementary material for: Imaging markers of cerebral small vessel disease are associated with Alzheimer’s disease: a systematic review and meta-analysis
Source: Front Aging Neurosci. 2025 Jan 28;17:1498636. doi: 10.3389/fnagi.2025.1498636 (PMC11894735; doi:10.3389/fnagi.2025.1498636)
Supplement: Supplementary file 1 [file Data_Sheet_1.docx]

**Search strategies for searching the MEDLINE (PubMed), Web of Science, Embase, and Cochrane database.**

**PUBMED 2980 Results**

**(((Cerebral Small Vessel Diseases[MeSH Terms]) OR (((((((((((((((((((((((White matter hyperintensities[Title/Abstract]) OR (white matter lesion[Title/Abstract])) OR (white matter disease[Title/Abstract])) OR (white matter change[Title/Abstract])) OR (leukoaraiosis[Title/Abstract])) OR (microbleed[Title/Abstract])) OR (microhemorrhage[Title/Abstract])) OR (microhaemorrhage[Title/Abstract])) OR (Lacunes[Title/Abstract])) OR (subcortical infarct[Title/Abstract])) OR (deep stroke[Title/Abstract])) OR (subcortical stroke[Title/Abstract])) OR (silent stroke[Title/Abstract])) OR (silent brain infarct[Title/Abstract])) OR (small vessel infarct[Title/Abstract] OR small vessel stroke[Title/Abstract])) OR (lacunar stroke[Title/Abstract])) OR (microinfarct[Title/Abstract])) OR (microscopic infarct[Title/Abstract])) OR (lacunar infarction[Title/Abstract])) OR (lacunar infarct[Title/Abstract])) OR (enlarged perivascular space[Title/Abstract])) OR (total imaging load[Title/Abstract])) OR (total burden[Title/Abstract]))) AND ((((Alzheimer Disease[MeSH Terms]) OR (Cognitive Dysfunction[MeSH Terms])) OR (Dementia[MeSH Terms])) OR ((((((((((((((((Alzheimer’s disease[Title/Abstract]) OR (amyloid beta-protein[Title/Abstract])) OR (Aβ[Title/Abstract])) OR (β-amyloid protein[Title/Abstract])) OR (Tau[Title/Abstract])) OR (Amyloid PET[Title/Abstract])) OR (Cognitive Disorder[Title/Abstract])) OR (Cognitive Impairment[Title/Abstract])) OR (Mild Cognitive Impairment[Title/Abstract])) OR (Impairment, Mild Cognitive[Title/Abstract])) OR (Cognitive Decline[Title/Abstract])) OR (Mental Deterioration[Title/Abstract])) OR (Amentia[Title/Abstract])) OR (Senile Paranoid Dementia[Title/Abstract])) OR (Paranoid Dementia, Senile[Title/Abstract])) OR (Familial Dementia[Title/Abstract])))) AND (((((((risk[MeSH Terms]) OR (relative risk[Title/Abstract])) OR (hazard ratio[Title/Abstract])) OR (OR[Title/Abstract])) OR (HR[Title/Abstract])) OR (RR[Title/Abstract]) OR (cohort[Title/Abstract])) OR (case-control[Title/Abstract]))**

**WOS 489 Results**

**(TS=(Cerebral Small Vessel Diseases) OR AB=(White matter hyperintensities OR white matter lesion OR white matter disease OR white matter change OR leukoaraiosis OR microbleed OR microhemorrhage OR microhaemorrhage OR Lacunes OR subcortical infarct OR deep stroke OR subcortical stroke OR silent stroke OR silent brain infarct OR small vessel infarct OR small vessel stroke OR lacunar stroke OR microinfarct OR microscopic infarct OR lacunar infarction OR lacunar infarct OR enlarged perivascular space OR total imaging load OR total burden)) AND (TS=(Alzheimer Disease OR Cognitive Dysfunction OR Dementia) OR AB=(Alzheimer’s disease OR amyloid beta-protein OR Aβ OR β-amyloid protein OR Tau OR Amyloid PET OR Amentia OR Senile Paranoid Dementia OR Paranoid Dementia, Senile OR Familial Dementia OR Cognitive Disorder OR Cognitive Impairment OR Mild Cognitive Impairment OR Impairment, Mild Cognitive OR Cognitive Decline OR Mental Deterioration OR Amentia OR Senile Paranoid Dementia OR Paranoid Dementia, Senile OR Familial Dementia)) AND (TS=(risk) OR AB=(relative risk OR hazard ratio OR HR OR RR OR cohort OR case-control))**

**Cochrane 483 Results**

**#1 MeSH descriptor: [Cerebral Small Vessel Diseases] explode all trees**

**#2 (Cerebral Small Vessel Diseases OR White matter hyperintensities OR white matter lesion OR white matter disease OR microbleed OR microhemorrhage OR microhaemorrhage OR Lacunes OR subcortical infarct OR lacunar stroke OR infarct OR lacunar infarction OR lacunar infarct OR enlarged perivascular space OR total imaging load OR total burden):ti,ab,kw**

**#3 MeSH descriptor: [Alzheimer Disease] explode all trees**

**#4 (Alzheimer’s disease OR amyloid beta-protein OR Aβ OR β-amyloid protein OR Tau OR Amyloid PET OR Cognitive Dysfunction OR Dementia OR Cognitive Disorder OR Cognitive Impairment OR Mild Cognitive Impairment OR Impairment, Mild Cognitive OR Cognitive Decline OR Mental Deterioration OR Amentia OR Senile Paranoid Dementia OR Paranoid Dementia, Senile OR Familial Dementia):ti,ab,kw**

**#5 MeSH descriptor: [Risk] explode all trees**

**#6 (relative risk or hazard ratio or RR or HR or Cohort or case-control):ti,ab,kw**

**#7 #1 OR #2**

**#8 #3 OR #4**

**#9 #5 OR #6**

**#10 #7 AND #8 AND #9**

**Embase 2535 Results**

**No. Query Results**

**#1. 'cerebrovascular disease'/exp OR 'cerebrovascular disease'**

**#2. 'white matter hyperintensities':ab,ti OR 'white matter lesion':ab,ti OR 'white matter disease':ab,ti OR 'white matter change':ab,ti OR leukoaraiosis:ab,ti OR microbleed:ab,ti OR microhemorrhage:ab,ti OR microhaemorrhage:ab,ti OR lacunes:ab,ti OR 'lacunar infarction':ab,ti OR 'lacunar infarct':ab,ti OR 'enlarged perivascular space':ab,ti OR 'total imaging load':ab,ti OR 'total burden':ab,ti**

**#3. 'alzheimer disease'/exp OR 'alzheimer disease'**

**#4. 'alzheimer disease':ab,ti OR 'amyloid beta-protein':ab,ti OR aβ:ab,ti OR 'β-amyloid protein':ab,ti OR tau:ab,ti OR 'amyloid pet':ab,ti OR 'cognitive disorder':ab,ti OR 'cognitive impairment':ab,ti OR 'mild cognitive impairment':ab,ti OR 'impairment, mild cognitive':ab,ti**

**#5. 'risk factor'/exp**

**#6. 'relative risk':ab,ti OR 'hazard ratio':ab,ti OR rr:ab,ti OR hr:ab,ti OR cohort:ab,ti OR 'case control study':ab,ti**

**#7. #1 OR #2**

**#8. #3 OR #4**

**#9. #5 OR #6**

**#10 #7 AND #8 AND #9**

| Supplementary Table I. Quality assessment of studies included in the meta-analyses | | | | | | | | |
| --- | --- | --- | --- | --- | --- | --- | --- | --- |
| Author, year | Sampling | Exposure ascertainment | Baseline screening | Adjustment | Outcome assessment | Follow-up  duration | Attrition | Overall  quality score |
| White matter hyperintensities | | | | | | | |  |
| Chen 2022 | 1 | 1 | 1 | 2 | 1 | 0 | 0 | 6 |
| Ding 2018  2012 | 1 | 1 | 1 | 2 | 1 | 1 | 0 | 7 |
| Miwa 2016 | 1 | 0 | 1 | 1 | 0 | 1 | 1 | 5 |
| Ye 2019 | 1 | 1 | 1 | 2 | 1 | 1 | 1 | 8 |
| Eckerström 2015 | 1 | 1 | 1 | 0 | 1 | 0 | 1 | 5 |
| Staekenborg 2009 | 1 | 1 | 1 | 1 | 1 | 0 | 1 | 6 |
| Rosano 2007  2016 | 1 | 1 | 1 | 2 | 1 | 0 | 1 | 7 |
| Tosto 2014 | 1 | 1 | 1 | 2 | 1 | 0 | 1 | 7 |
| Keller 2023 | 1 | 1 | 1 | 1 | 1 | 1 | 1 | 7 |
| Cerebral microbleeds | | | | | | | |  |
| Akoudad 2016  Ding 2018  (2017)  2003 | 1 | 1 | 1 | 2 | 1 | 0 | 1 | 7 |
| Ding 2018  Miwa 2014 | 1 | 1 | 1 | 2 | 1 | 1 | 1 | 6 |
| Miwa 2014 | 1 | 1 | 1 | 1 | 1 | 1 | 1 | 7 |
| Romero 2017 | 1 | 1 | 1 | 1 | 1 | 1 | 1 | 7 |
| Staekenborg2009 | 1 | 1 | 1 | 1 | 1 | 1 | 1 | 7 |
| Lacunar infarctions | | | | | | | |  |
| Ding 2018  2016 | 1 | 1 | 1 | 2 | 1 | 1 | 1 | 8 |
| Miwa 2016 | 1 | 0 | 1 | 1 | 0 | 1 | 1 | 5 |
| Rosano 2007 | 1 | 1 | 1 | 2 | 1 | 0 | 1 | 7 |
| Staekenborg 2009 | 1 | 1 | 1 | 1 | 0 | 0 | 1 | 5 |

**Supplementary Table I. (continued)**

| Author, year | | Sampling | Exposure ascertainment | | Baseline screening | | Adjustment | Outcome assessment | | | Follow-up  duration | Attrition | Overall  quality score |
| --- | --- | --- | --- | --- | --- | --- | --- | --- | --- | --- | --- | --- | --- |
| Ye 2019 | | 1 | 1 | | 1 | | 2 | 1 | | | 1 | 1 | 8 |
| Enlarged perivascular spaces | | | | | | | | | | | | |  |
| Chen 2022  Paradise 2021 | 1 | | | 1 | | 1 | 2 | | 1 | 0 | | 1 | 7 |
| Paradise 2021  Romero,J 2022 | 1 | | | 1 | | 1 | 2 | | 1 | 1 | | 1 | 8 |
| Romero, J 2022  Shi 2023 | 1 | | | 1 | | 1 | 2 | | 1 | 1 | | 1 | 8 |
| Shi 2023 | 1 | | | 1 | | 1 | 2 | | 1 | 1 | | 1 | 8 |

Scoring criteria were defined as follows: Sampling: 1 = random sampling from the population, 0 = selected group or not described; Exposure ascertainment for white matter hyperintensities: 1 = (semi)quantitative, 0 = qualitative, for lacunar infarctions: 1=standard definition >= 3 mm, 0 = other; Baseline screening for outcome: 1 = yes, 0 = no; Adjustment: 2 = adjusted for most potential confounders, 1 = age and sex adjusted only, 0 = unadjusted; Outcome assessment: 1 = independent blind assessment or record linkage, 0 = self-report or not described; Follow-up duration: 1 = adequate (≥5 years), 0 = short (<5 years); Attrition: 1 = <20%, 0 = ≥20%; Overall quality score: sum of the scores of the individual quality criteria (range 0 to 8)

**Figure S1** sensitivity analysis of white matter hyperintensities.

**Figure S2** sensitivity analysis of cerebral microbleeds

**Figure S3** sensitivity analysis of lacunar infarctions

**Figure S4** Sensitivity analysis of enlarged perivascular spaces

**Figure S5** Forest plots of subgroups of white matter hyperintensities by **Assessment Methods.**


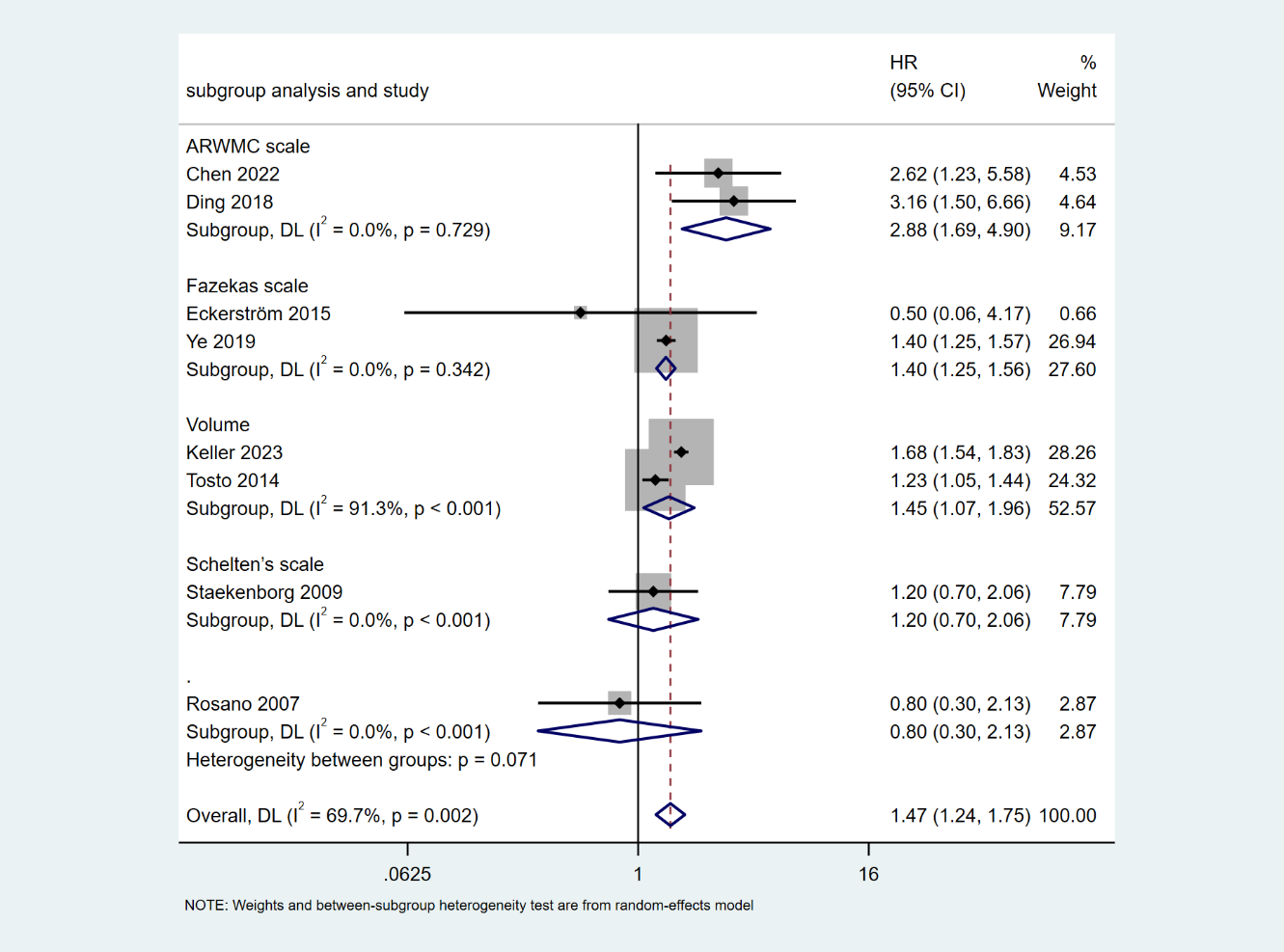


HR = hazard ratio.

**Figure S6** Forest plots of subgroups of white matter hyperintensities by **MRI sequences.**


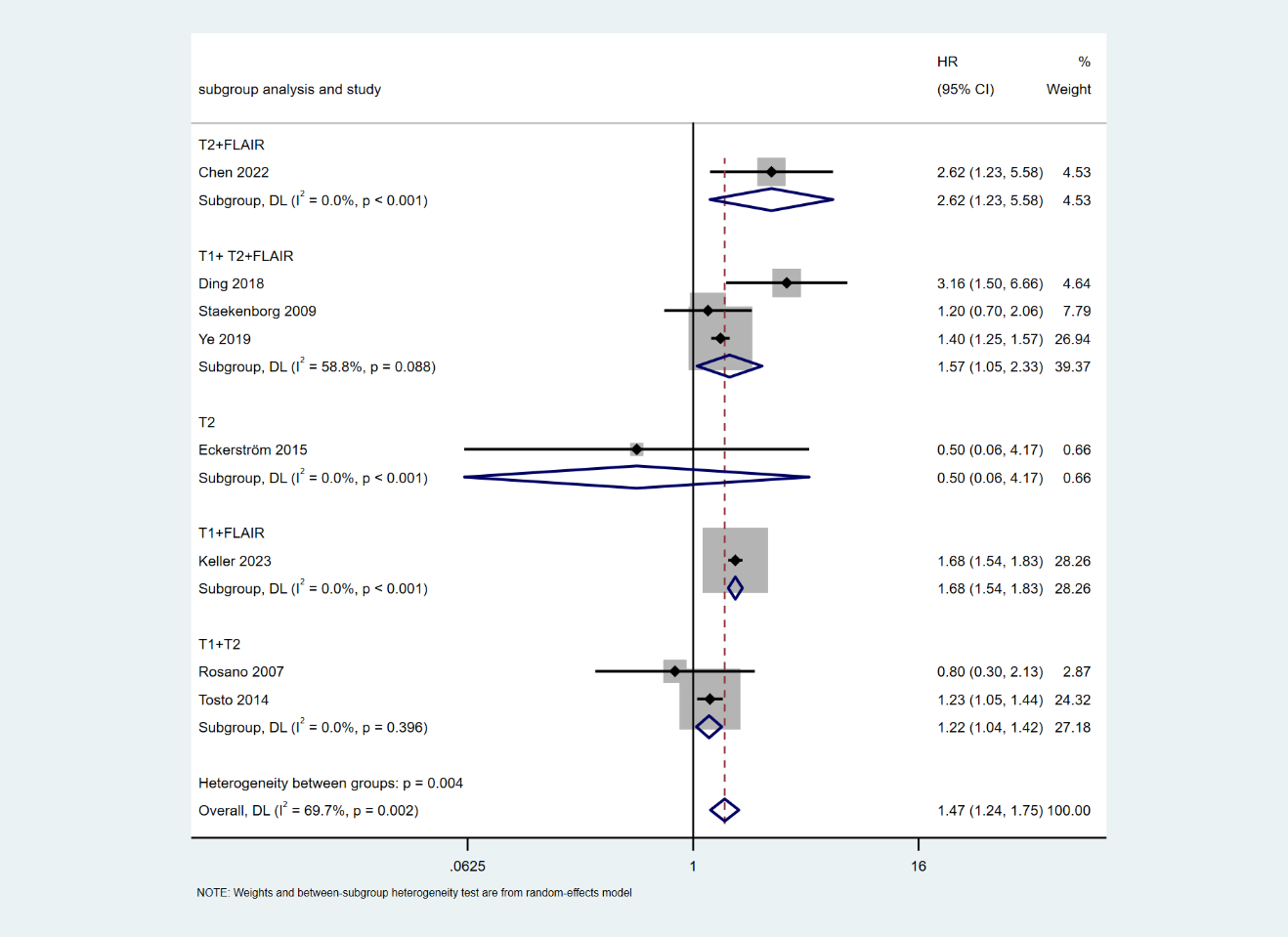


HR = hazard ratio.
